# MicroRNA‐524‐5p suppresses the progression of papillary thyroid carcinoma cells via targeting on FOXE1 and ITGA3 in cell autophagy and cycling pathways

**DOI:** 10.1002/jcp.28472

**Published:** 2019-04-02

**Authors:** Hui Liu, Xi Chen, Ting Lin, Xingsheng Chen, Jiqi Yan, Shan Jiang

**Affiliations:** ^1^ Key Laboratory of Stem Cell Engineering and Regenerative Medicine, Department of Human Anatomy, Histology and Embryology School of Basic Medical Sciences, Fujian Medical University Fuzhou Fujian China; ^2^ Department of Surgery Ruijin Hospital Affiliated of Shanghai Jiaotong University School of Medicine Shanghai People's Republic of China; ^3^ Department of Vascular Thyroid Surgery Affiliated Union Hospital, Fujian Medical University Fuzhou Fujian China

**Keywords:** FOXE1, ITGA3, microRNA, miR‐524‐5p, papillary thyroid carcinoma

## Abstract

MicroRNAs are beneficial for cancer therapy as they can simultaneously downregulate multiple targets involved in diverse biological pathways related to tumor development. In papillary thyroid cancer, many microRNAs were identified as differentially expressed factors in tumor tissues. In another way, recent studies revealed cell proliferation, cell cycling, apoptosis, and autophagy are critical pathways controlling papillary thyroid cancer development and progression. As miR‐524‐5p was approved as a cancer suppressor targeting multiple genes in several types of cancer cells, this study aims to characterize the role of miR‐524‐5p in the thyroid cancer cell. The expression of miR‐524‐5p was decreased in the papillary thyroid cancer tissues and cell lines, while forkhead box E1 (FOXE1) and ITGA3 were increased. In the clinical case, expression of miR‐524‐5p, FOXE1, and ITGA3 were significantly correlated with papillary thyroid cancer development and progression. FOXE1 and ITGA3 were approved as direct targets of miR‐524‐5p. miR‐524‐5p could inhibit papillary thyroid cancer cell viability, migration, invasion, and apoptosis through targeting FOXE1 and ITGA3. Cell cycling and autophagy pathways were disturbed by downregulation of FOXE1 and ITGA3, respectively. Collectively, miR‐524‐5p targeting on FOXE1 and ITGA3 prevents thyroid cancer progression through different pathways including cell cycling and autophagy.

## 1 INTRODUCTION

Thyroid cancer is diagnosed 1% in the proportion of all tumors detected by clinical in developing countries (Lacin, Esin, Karakas, & Yalcin, 2015). It is suggested that the endocrine system has the highest cancerization risk in the thyroid with an increased tendency each year. Thyroid cancer has multiple subtypes, including undifferentiated, medullary, follicular, and papillary thyroid cancer (PTC). PTC is the most common thyroid malignancy with 80% in the proportion of all thyroid malignancies. The follicular cell is the origin of PTC (Dalal, Kaur, & Bansal, 2017; Hundahl, Fleming, Fremgen, & Menck, 1998; Tata et al., 2018). It was well established that epithelial–mesenchymal transition (EMT) is one of the most crucial steps in PTC development (T. Chen, You, Jiang, & Wang, 2017). During EMT, the epithelial cell gets mesenchymal characteristics instead of it's own. It is implicated that tumor invasion and metastasis require this transition which allows polarized epithelial cells to get access to move in the cellular matrixes. Cancer‐cell proliferation and cell‐cycle progression were also involved in the PTC progression. After decades of studies in molecular biology, researchers have identified several oncogenes related proliferation and cell cycle of cancer cell, such as LCN2, CRABP1, C1QL1, PTEN, and BRAF (Celestino et al., 2018; Loo, Khalili, Beuhler, Siddiqi, & Vasef, 2018; Razavi, Modarressi, Yaghmaei, Tavangar, & Hedayati, 2017). In recent years, it was implicated that autophagy has a vital role in PTC development and progression. Without FAM129A induced autophagy in normal thyroid cells, carcinomas are developed under certain nutrient and growth condition (Nozima et al., 2019). miR‐125b could enhance autophagy in PTC through repression of Foxp3 (Wang et al., 2018). miR‐144 reduces the tumor growth by suppressing transforming growth factor‐α signaling pathway to promote autophagy of PTC (J. Liu et al., 2018). Obviously, targets for PTC therapy exist in multiple biological pathways. Both EMT and autophagy attracted researchers' attention to survey underline mechanisms related to PTC development and progression.

In the complicated network of carcinoma development, microRNAs (miRNAs) have the critical role as they can simultaneously downregulate multiple targets (Dallaire, Frederick, & Simard, 2018; Fitzwalter et al., 2018; Kasper et al., 2017; Singh, Lee, Hartman, Ruiz‐Whalen, & O'Reilly, 2018). Thus, it is possible to modify cell proliferation, cycling, and autophagy in carcinoma cells at the same time though controlling miRNAs expression. In PTC, there are several differentially expressed miRNAs were identified by miRNA sequencing (Graham et al., 2015). PTC cell proliferation is prevented by miR‐7 targetingCDC28 protein kinase regulatory subunit 2 (CKS2) (Hua et al., 2016). This interaction also influences on the migration and invasion of PTC. Whereas, miR‐150‐5p targeting on BRAFV600E mutation could accelerate PTC development by enhancing EMT (Yan et al., 2018). During the processes, the PTC cell proliferation and apoptosis are also affected by miR‐150‐5p with regulation on BRAFV600E. The typical tumor pathway AKT/mTOR in PTC development and progression is also regulated by miR‐451a (Minna et al., 2016). To turn down the activation of AKT/mTOR pathway, multiple targets including AKT1, MIF, and c‐MYC are downregulated by miR‐451a. Attenuated AKT/mTOR signaling pathway through ectopic expression of miR‐451a could damage the processes of PTC cell proliferation and migration. Another typical miRNA function as a tumor suppressor in PTC is let‐7 miRNA targeting on RAS. Recent studies suggested that the let‐7 family from circulating miRNA could be served as a clinical marker to noninvasively diagnose PTC and prognose survival after treatment (Perdas, Stawski, Nowak, & Zubrzycka, 2016).

Using developed sequencing biotechnology on clinical samples, more miRNAs were determined as differentially expressed miRNAs in the PTC compared with normal thyroid tissue. There is still unknown mechanism effecting PTC development and progression related to miRNA remained to be studied. miR‐524‐5p that has been demonstrated as a tumor suppressor in some types of cancer cells, is not yet analyzed in PTC. The cell proliferation and migration of gastric cancer cells and pituitary tumor‐derived cell line are retarded by miR‐524‐5p (G. H. Liu, Liu, Yang, Zhu, & Zhao, 2016; Zhen et al., 2017). The growth of glioma is decreased by miR‐524‐5p targeting on EZH2, Jagged‐1, and Hes‐1 (L. Chen et al., 2012; Zhi et al., 2017). Oncogene BRAF and ERK2 could be downregulated by miR‐524‐5p in melanoma to reduce tumor growth (S. M. Liu, Lu, Lee, Chung, & Ma, 2014). However, the role of miR‐524‐5p is still unclear in PTC where miR‐524‐5p was already detected with differential expression level.

In this study, we investigated the relationship between miR‐524‐5p and PTC development and uncovered the underlying mechanisms by identification of its major targeting genes and biological pathway.

## 2 MATERIALS AND METHODS

### 2.1 Patients and tissue sample

PTC and para‐PTC tissues were received from Affiliated Union Hospital of Fujian Medical University (Fuzhou, China) and stored at −80°C. All experiments in this study were approved by the Ethics Committee of the Affiliated Union Hospital of Fujian Medical University. Before studies, informed consents were signed by all patients providing samples in this study. All patients were diagnosed and examined by the Affiliated Union Hospital of Fujian Medical University (Fuzhou, China) with at least two pathologists.

### 2.2 Cell lines

Stem Cell Bank, Chinese Academy of Sciences (Shanghai, China) provided the TPC‐1, K1, and NPA cell lines. The growth condition followed the manufacturer's instructions and incubated at 37°C in Roswell Park Memorial Institute (RPMI)‐1640 medium containing 10% fetal bovine serum.

### 2.3 RNA extraction and isolation

TRIzol Reagent (Thermo Fisher Scientific, Waltham, MA), NanoDrop ND‐1000 spectrophotometer (Thermo Fisher Scientific) and the Agilent RNA 6000 (Thermo Fisher Scientific) Nano assay was utilized to isolate the total RNA and assess the RNA quantity, purity, and integrity. The experiments performed following the manufacturer's instructions. Gel electrophoresis was used to detect genomic DNA contamination. RNA with A260/A280 ≥ 1.6 and A260/A230 ≥ 1 were used for the following experiments.

### 2.4 Real‐time quantitative PCR (RT‐qPCR)

The complementary DNA was produced by the Prime Script™ RT‐qPCR Kit (Thermo Fisher Scientific, Inc.) following the manufacturer's instructions. The quantification PCR were carried out in 7900HT fast RT‐qPCR instrument (Applied Biosystems, Life Technologies, Cologne, Germany) with SYBR® Premix Ex Taq™ II (Takara Bio Inc., Tokyo, Shiga, Japan). The endogenous control was the expression of glyceraldehyde‐3‐phosphate dehydrogenase was used as the control. The level of genes were calculated by the $2^{-\Delta \Delta C_{t}}$ method. All qPCR was performed in triplicate.

### 2.5 Western blot analysis

Cells were lysed in radioimmunoprecipitation assay (RIPA) buffer (Takara Bio Inc.) added a complete protease inhibitor cocktail after washing with phosphate‐buffered saline (PBS). The protein concentration was accessed by dye reagent in Bio‐Rad Protein Assay (Bio‐Rad Laboratories, Hercules, CA) with standard bovine serum albumin. After separation by sodium dodecyl sulfate polyacrylamide gel electrophoresis, proteins were transferred to polyvinylidene difluoride membranes. Blocking solution (20 mM Tris–HCl, 0.1% Tween‐20, 5% nonfat‐milk, and 150 mM NaCl) was applied on membranes for 2 hr at room temperature. After three times phosphate‐buffered saline with Tween 20 (PBST) buffer washing, primary antibodies were applied for 2 hr incubation. Next four times PBST buffer washing, the secondary antibody were added for overnight incubation. Next four times PBST buffer washing, Enhanced Chemiluminescence Detection Kit (KGP116, KeyGen BioTECH, Nanjiang, China) was utilized for detection of blots.

### 2.6 Luciferase reporter assay

3′ untranslated region (3′UTR) of matrilin 2 (MATN2), forkhead box E1 (FOXE1), and ITGA3 including the predicted miR‐524‐5p binding site was amplified and constructed into the psiCHECK‐2 reporter vector (Promega, Madison, WI). Also, mutant version was produced. After cotransfection with miR‐524 mimics, Luciferase activity was determined by Dual‐Luciferase Reporter Assay Kit (Promega).

### 2.7 Cell transfection

miR mimics and inhibitor, RNAi were generated by Shanghai Jima Co., Ltd. (Shanghai, China). The experiments were performed using Lipofectamine 2000 (Invitrogen, Carlsbad, CA) following the manufacturer's instruction. Cell Counting Kit‐8 Reagent (Beyotime Institute of Biotechnology, Shanghai, China)

### 2.8 3‐(4,5‐Dimethythiazol‐2‐yl)‐2,5‐diphenyl tetrazolium bromide (MTT) migration and invasion assays

Cell Counting Kit‐8 Reagent (Beyotime Institute of Biotechnology) was used to cell proliferation assay. In colony formation assay, cell was grown in six‐well transwell plates for 2 weeks. Crystal violet solution (0.5%) was used to stained the positive cells after fixation. For scratch test, A 10 μl pipet tip was used to make lines and after certain hours, cells were imaged and determine the distance of gap. All assays were carried out biological independently in triplicate.

### 2.9 Cell cycle and apoptosis analysis

After washing with PBS, cells were fixed in 75% ethanol at 4°C overnight. Again washed with PBS, propidium iodide was used to stain cell in the dark at 37°C. Flow Cytometry (Beckman Coulter, Inc., Brea, CA) was used to measure the cell populations in different phases. To determine the cell apoptosis, Annexin V‐FITC Kit (Becton, Dickinson and Company, Franklin Lakes, NJ) was used according to the manufacturer's protocol. Finally, Flow cytometry (Beckman Coulter, Inc.) was used to measure the cell populations in apoptosis. All assays were carried out biological independently in triplicate.

### 2.10 Statistical analysis

SPSS 21.0 Software (SPSS version 21.0., IBM Corp., Armonk, NY) and GraphPad Prism 5 (GraphPad Software Inc., San Diego, CA) were used to do statistical analyses and graphing. All data were shown as mean ± standard deviation. The differences between groups were tested by Student's *t* test and analysis of variance. The significant difference was considered when *p* < 0.05.

## 3 RESULTS

### 3.1 miR‐524‐5p is downregulated while *FOXE1* and *ITGA3* are upregulated in PTC tissues

**Figure 1:**
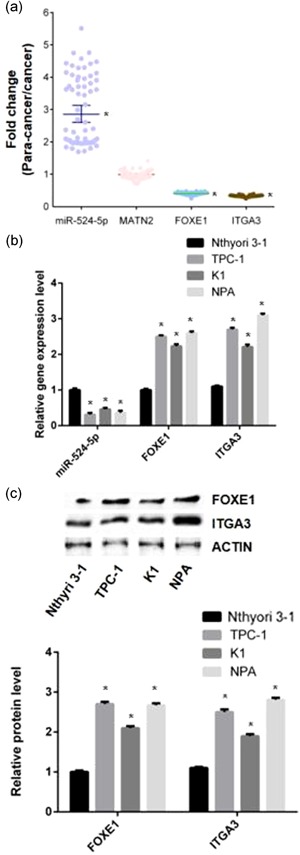
The expression of miR‐524‐5p is the association with FOXE1 and ITGA3 in PTC tissues. (a) The expression of miR‐524‐5p, MATN2, FOXE1, and ITGA3 in PTC comparing to papa‐cancer tissues. (b) The messenger RNA levels of miR‐524‐5p, FOXE1, and ITGA3 were determined by quantitative reverse‐transcription polymerase chain reaction in normal thyroid cell (Nthyori 3‐1) and three types of PTC cell lines (TPC‐1, K1, and NPA). (c)The FOXE1 and ITGA3 protein expression levels were determined by western blot analysis in normal thyroid cell (Nthyori 3‐1) and three types of PTC cell lines (TPC‐1, K1, and NPA). Actin was detected as a loading control. FOXE1: forkhead box E1; MATN2: matrilin 2; PTC: papillary thyroid cancer [Color figure can be viewed at wileyonlinelibrary.com]

To determine the miR‐524‐5p expression in cancer and papa‐cancer tissues, RT‐qPCR was carried out with tissues from 57 PTC patients. Results displayed that expression of miR‐524‐5p in PTC was lower than adjacent normal tissues with a significant fold change < 0.3 (Figure 1a). The potential targets of miR‐524‐5p were predicted by the online software. According to the prediction results, *MATN2*, *FOXE1*, and *integrin subunit alpha 3* (*ITGA3*) are the target gene of miR‐524‐5p with the specific binding regions in 3′UTR. RT‐qPCR was utilized to detect the expression pattern of *MATN2*, *FOXE1*, and *ITGA3* in PTC and papa‐cancer tissues. The *FOXE1* and *ITGA3* were significantly raised to 2.5 and three times in PTC comparing to papa‐cancer tissues (Figure 1a). No significant change was found in *MATN2* expression between PTC and papa‐cancer tissues (Figure 1a).

### 3.2 The expression level of miR‐524‐5p, *FOXE1*, and *ITGA3* in normal thyroid cell is different from PTC cell

To compare the expression of miR‐524‐5p, *FOXE1*, and *ITGA3* between normal thyroid cell line (Nthyori 3‐1) and three types of PTC cell lines (TPC‐1, K1, and NPA), RT‐qPCR was applied to each cell line. Results show that expression of miR‐524‐5p was significantly reduced in TPC‐1, K1, and NPA compared with Nthyori 3‐1 (Figure 1b). The fold change of miR‐524‐5p expression is lowest in K1 and highest in TPC‐1. Increased expression of *FOXE1* and *ITGA3* was found in TPC‐1, K1, and NPA compared with Nthyori 3‐1 (Figure 1b). Western blot analysis was carried out to investigate the protein level of *FOXE1* and *ITGA3*. As results displayed, *FOXE1* and *ITGA3* have more higher protein level in the PTC cell lines than the normal cell line (Figure 1c).

### 3.3 The progression of PTC is associated with the level of miR‐524‐5p

The clinical characteristics of PTC patients were collected for further analysis of the relationship between the PTC progression and miR‐524‐5p, *FOXE1*, or *ITGA3* expression. According to the age, gender, tumor size, degree of differentiation, lymph node metastases (LNM), degree of invasion, and TNM stage, patients were grouped and averaged expression levels of miR‐524‐5p was used to compare differences (Table 1). The significantly lower levels of miR‐524‐5p were detected in LNM, intrathyroid carcinoma and I‐II TNM stages (*p* < 0.05).

**Table 1:** The relationship between the pathological features and relative expression of miR‐524‐5p (para‐cancer/cancer) in thyroid cancer patients

Pathological features	miR‐524‐5p	*p*
Age		
<45	2.82 ± 0.17	0.471
≥45	2.72 ± 0.14	
Gender		
Female	2.74 ± 0.12	0.527
Male	2.79 ± 0.13	
Degree of differentiation		
Low	2.87 ± 0.22	0.428
Middle high	2.62 ± 0.19	
Lymph node metastases		
Without	2.13 ± 0.23	0.003
With	3.78 ± 0.32	
Degree of invasion		
Extrathyroid	2.21 ± 0.21	0.002
Intrathyroid	3.69 ± 0.27	
TNM		
I–II	3.71 ± 0.31	0.005
III–IV	2.17 ± 0.34	

### 3.4 *FOXE1* and *ITGA3* are target genes of miR‐524‐5p in PTC cells

**Figure 2:**
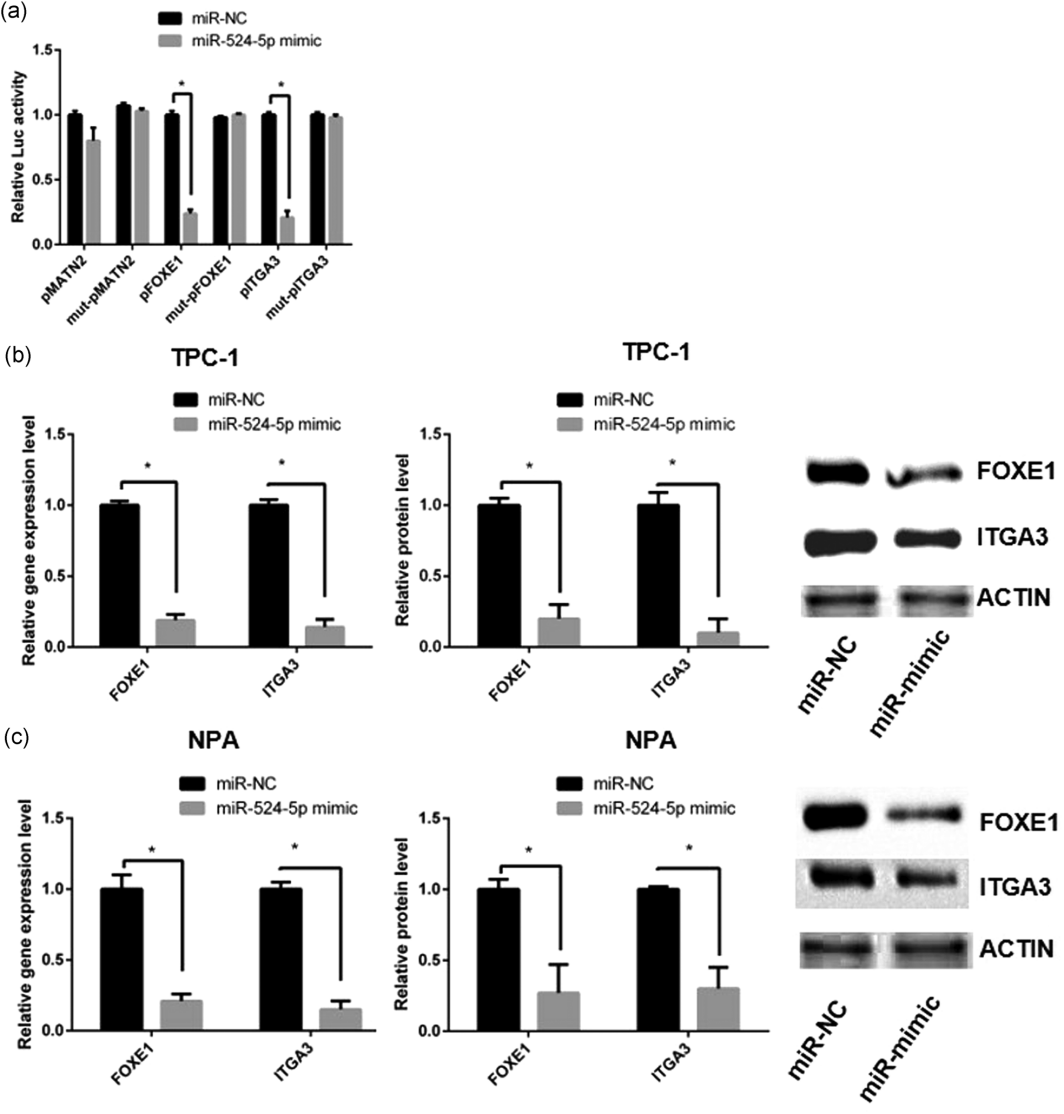
FOXE1 and ITGA3 are the direct targets of miR‐524‐5p in PTC cells. (a) The luciferase activity of MATN2, FOXE1, and ITGA3. The messenger RNA and protein expression levels of FOXE1 and ITGA3 were detected in TPC‐1 (b) and in NPA cells (c). FOXE1: forkhead box E1; MATN2: matrilin 2; PTC: papillary thyroid cancer

To prove the predicted target genes of miR‐524‐5p, dual‐luciferase reporter gene assays were performed using wide‐type and mutated 3′UTR of *MATN2*, *FOXE1*, and *ITGA3*. Results showed miR‐524‐5p mimic significantly knockdown luciferase activity of wide‐type *FOXE1* and *ITGA3* 3′UTR groups, while mutant FOXE1 and ITGA3 3′UTR groups recovered the luciferase activity. No significant change was detected in both wide‐type and mutated 3′UTR of *MATN2*. The result indicated that miR‐524‐5p could interact with *FOXE1* and *ITGA3* 3′UTR to knockdown the expression (Figure 2a).

To confirm the downregulation of miR‐524‐5p on *FOXE1* and *ITGA3* in PTC, miR‐524‐5p mimic was added into PTC cell lines including TPC‐1 and NPA. The messenger RNA (mRNA) and protein levels of *FOXE1* and *ITGA3* were detected by RT‐qPCR and western blot analysis. Results demonstrated that both mRNA and protein levels of *FOXE1* and *ITGA3* were significantly decreased by miR‐524‐5p mimic in TPC‐1 and NPA cells (Figure 2b,c). All results indicate that *FOXE1* and *ITGA3* are target genes of miR‐524‐5p in PTC cells.

### 3.5 TPC‐1 and NPA cell viabilities were downregulated by miR‐524‐5p, siRNA‐FOXE1, or siRNA‐ITGA3

**Figure 3:**
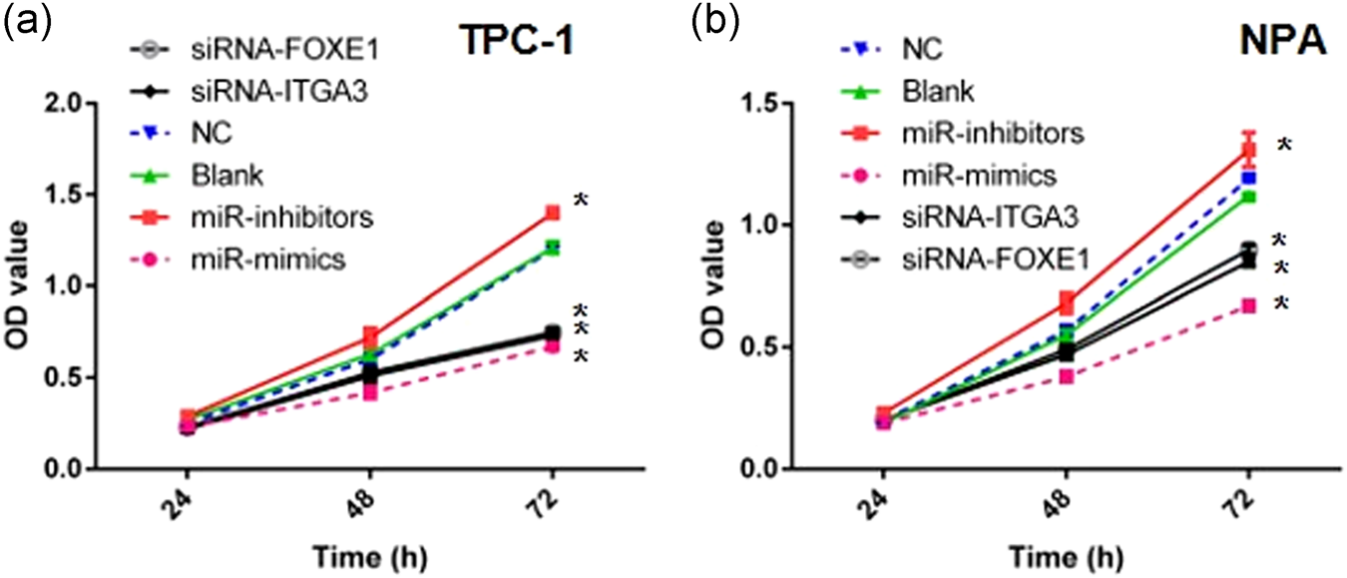
Cell viability of TPC‐1 (a) and NPA (b) cells with certain preprocesses were determined by MTT assay. miR‐524‐5p mimic groups showed decreased cell viability in both TPC1 and NPA cells (*p* < 0.05), while miR‐524‐5p inhibitor groups increased. siRNA‐FOXE1 and siRNA‐ITGA3 groups have reduced cell viabilities (*p* < 0.05). FOXE1: forkhead box E1; MTT: 3‐(4,5‐dimethythiazol‐2‐yl)‐2,5‐diphenyl tetrazolium bromide; siRNA: small interfering RNA [Color figure can be viewed at wileyonlinelibrary.com]

Cell viability of TPC‐1 and NPA with certain preprocesses were determined by MTT assay. Results displayed no significant difference among the NC and blank groups in both TPC‐1 and NPA cells (Figure 3a,b). Both TPC‐1 and NPA cell viabilities of miR‐524‐5p mimic groups were significantly decreased (*p* < 0.05), while miR‐524‐5p inhibitor groups had significantly increased cell viability in comparison with the NC and blank groups (*p* < 0.05; Figure 3a,b). Compared with blank and NC groups, siRNA‐FOXE1, and siRNA‐ITGA3 groups also showed significantly reduced TPC1 and NPA cell viabilities (*p* < 0.05; Figure 3a,b). In addition, both siRNA‐FOXE1 and siRNA‐ITGA3 groups in NPA cells had significantly higher cell viability than miR‐524‐5p mimic groups (*p* < 0.05; Figure 3a,b). All results revealed that TPC‐1 and NPA cell viabilities were downregulated by miR‐524‐5p and upregulated by *FOXE1* or *ITGA3*.

### 3.6 PTC cell migration and invasion were suppressed by miR‐524‐5p, siRNA‐FOXE1, or siRNA‐ITGA3

**Figure 4:**
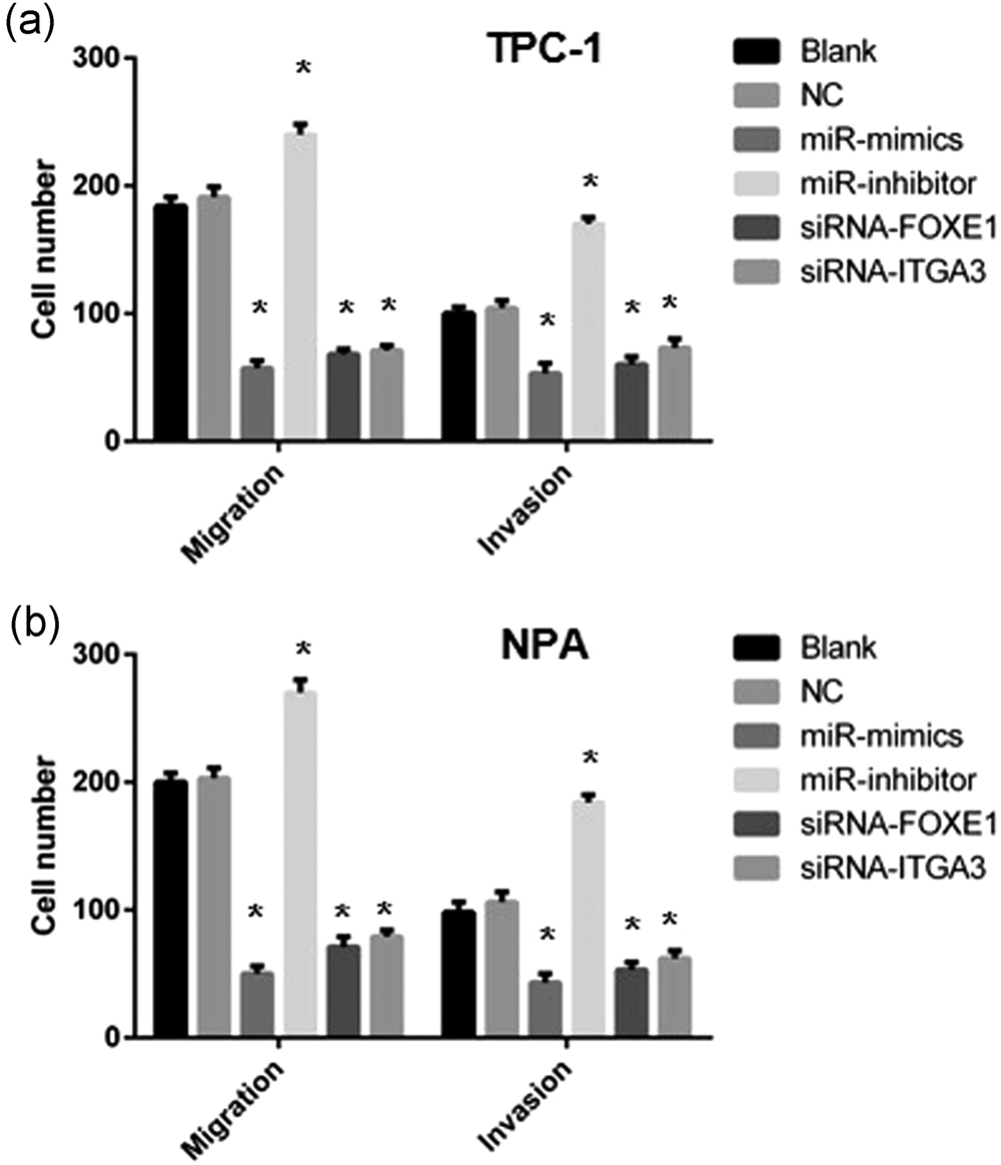
Cell migration and invasion of miR‐524‐5p, FOXE1, and ITGA3 in TPC‐1 (a) and NPA (b) cells were determined by scratch test and transwell assay. miR‐524‐5p mimic group showed decreased numbers of migration and invasion cells, while miR‐524‐5p inhibitor groups showed significantly increased numbers. Both siRNA‐FOXE1 and siRNA‐ITGA3 groups showed reduced cell numbers (*p* < 0.05). FOXE1: forkhead box E1; siRNA: small interfering RNA

To evaluate the role of miR‐524‐5p, *FOXE1*, and *ITGA3* in the TPC‐1 cell invasion and migration, TPC‐1 transfected with NC, miR‐524‐5p mimic, inhibitor, siRNA‐FOXE1, or siRNA‐ITGA3 were carried out by scratch test and transwell assay. There is no significant change in cell migration and invasion abilities between the NC and blank groups (Figure 4a). Compared with the NC group, the miR‐524‐5p mimic group has significantly decreased numbers of migration and invasion cells, while miR‐524‐5p mimic groups showed significantly increased numbers (*p* > 0.05; Figure 4a). In comparison with the NC group, the numbers of migration and invasion TPC‐1 cells were decreased in both siRNA‐FOXE1 and siRNA‐ITGA3 groups (*p* < 0.05; Figure 4a). In NPA cell, miR‐524‐5p mimic, inhibitor, siRNA‐FOXE1 and siRNA‐ITGA3 demonstrated a similar effect on the number of cell migration and invasion (Figure 4b).

### 3.7 PTC cell‐cycle arrest was enhanced by miR‐524‐5p or siRNA‐ITGA3

**Figure 5:**
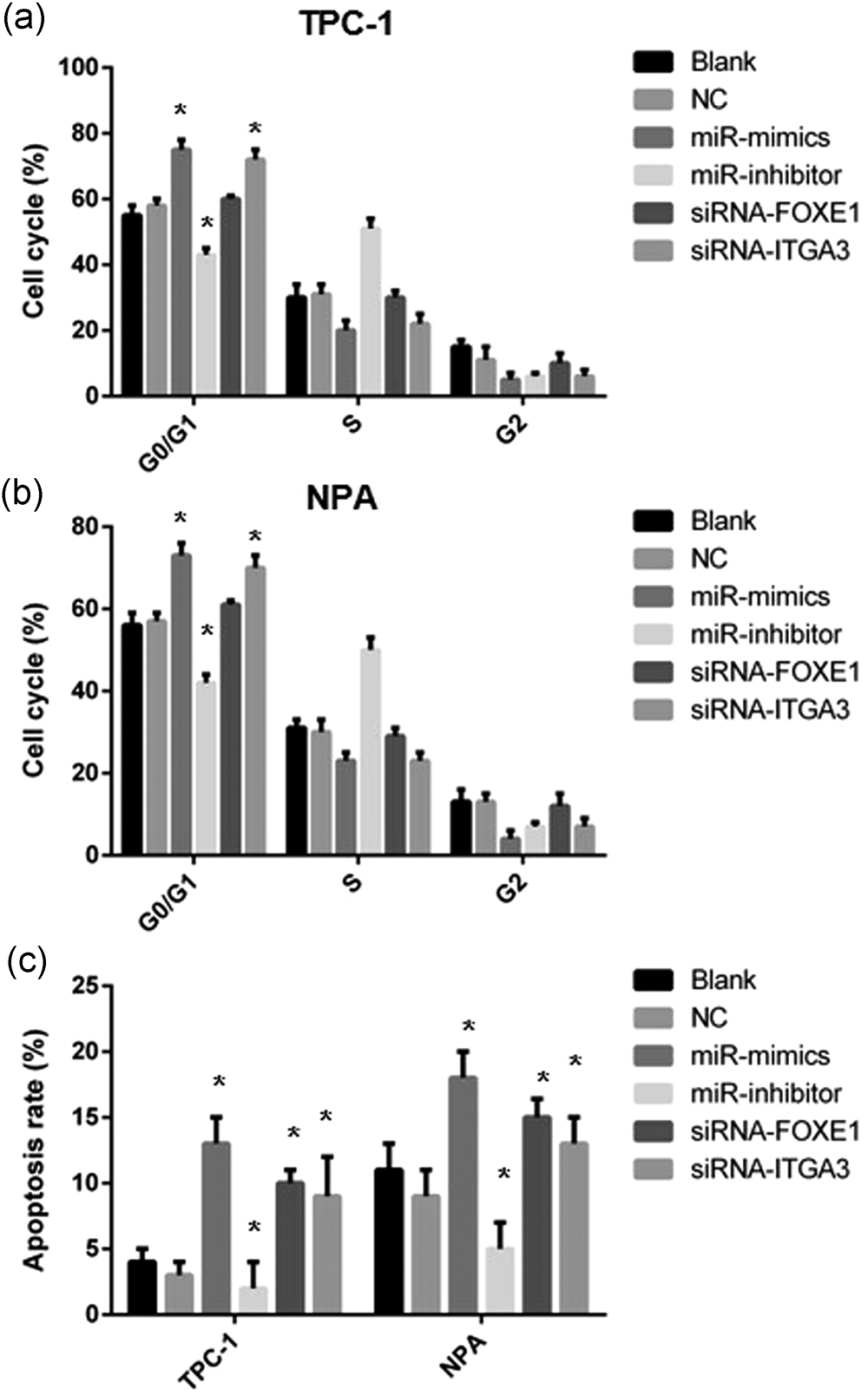
Cell cycle and apoptosis of miR‐524‐5p, siRNA‐FOXE1 or siRNA‐ITGA3. The cell‐cycle arrest was measured by flow cytometry in TPC‐1 (a) and NPA (b) cells. (c) Cell apoptosis of miR‐524‐5p, siRNA‐FOXE1, or siRNA‐ITGA3 in TPC‐1 and NPA was determined by flow cytometry. FOXE1: forkhead box E1; siRNA: small interfering RNA

To investigate the role of miR‐524‐5p, *FOXE1*, and *ITGA3* in PTC cell cycle, TPC‐1 and NPA transfected with NC, miR‐524‐5p mimic, inhibitor, siRNA‐FOXE1 or siRNA‐ITGA3 were measured by flow cytometry. Percentage of G0/G1 phase TPC‐1 and NPA cells was significantly increased in miR‐524‐5p mimic groups whereas significantly decreased in miR‐524‐5p inhibitor groups compared with the NC and blank groups (*p* < 0.05; Figure 5a,b). The proportion of the S phase TPC‐1 and NPA cells were reversely changed in miR‐524‐5p mimic and inhibitor groups (*p* < 0.05; Figure 5a,b). Proportion of G0/G1 phase and the S phase TPC‐1 and NPA cells in siRNA‐ITGA3 group were increased and decreased, respectively (*p* < 0.05; Figure 5a,b). Other groups had no significant differences in the pattern of cell cycle (Figure 5a,b).

### 3.8 PTC cell apoptosis was enhanced by miR‐524‐5p, FOXE1, or siRNA‐ITGA3

To survey the effect of miR‐524‐5p, *FOXE1*, and *ITGA3* on PTC cell apoptosis, apoptosis rate of TPC‐1 and NPA under different transfection were determined by flow cytometry. Results showed that the apoptosis rate of TPC‐1 and NPA was significantly increased in miR‐524‐5p mimic groups (Figure 5c). miR‐524‐5p inhibitor groups in NPA significantly reduce the apoptosis rate (Figure 5c). Both siRNA‐FOXE1 and siRNA‐ITGA3 groups had increased apoptosis rate in apoptosis rate (Figure 5c).

### 3.9 Autophagy‐related protein and cycle cyclin‐dependent kinase are regulated by miR‐524‐5p

**Figure 6:**
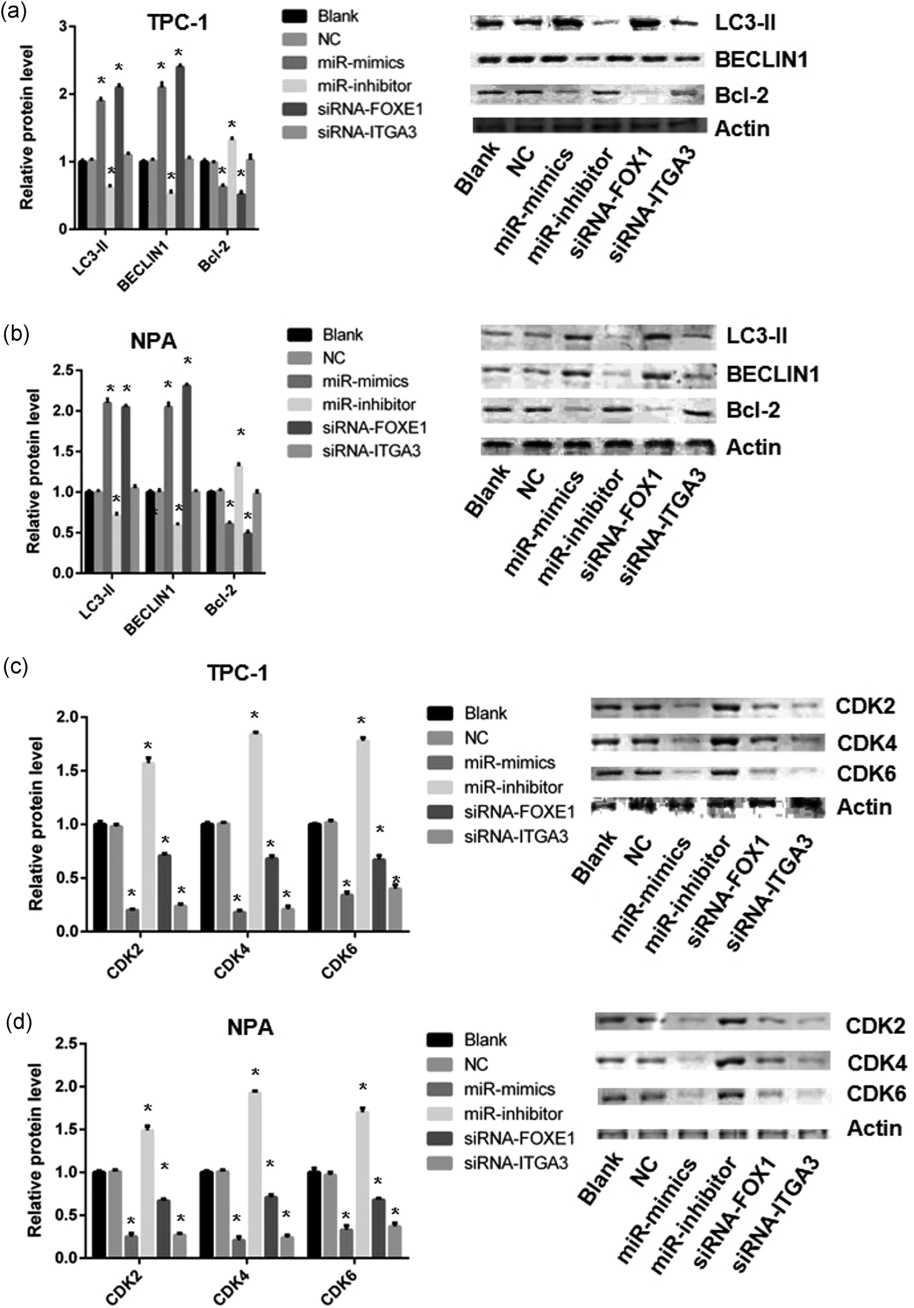
Effect of miR‐524‐5p on TPC‐1 and NPA cells. (a, b) Protein expression of LC3‐II, BECLIN1, Bcl‐2, FOXE1, and ITGA3 in TCP‐1 and NPA cells. (c, d) Protein expression of CDK2, CDK4, and CDK6 in TCP‐1 and NPA cells. Bcl‐2: B‐cell lymphoma‐2; CDK: cyclin‐dependent kinase; FOXE1: forkhead box E1; siRNA: small interfering RNA

To analysis the relationship between autophagy and miR‐524‐5p, autophagy‐related protein level was determined by western blot analysis in TPC‐1 and NPA under different transfection. Results showed that LC3‐II and BECLIN1 were significantly increased in miR‐524‐5p mimic group while decreased in miR‐524‐5p inhibitor group (Figure 6a,b). siRNA‐FOXE1 group has the similar result of LC3‐II and BECLIN1 to miR‐524‐5p mimic group with downregulated FOXE1 protein level (Figure 6a,b). B‐cell lymphoma‐2 (Bcl‐2) was decreased in miR‐524‐5p mimic and siRNA‐FOXE1 groups, while increased in miR‐524‐5p inhibitor group (Figure 6a,b). siRNA‐ITGA3 group shows no change on LC3‐II, BECLIN1, and Bcl‐2 with downregulated ITGA3 protein level (Figure 6a,b).

Protein level of CDK2, CDK4, and CDK6 were compared by western blot analysis in TPC‐1 and NPA under different transfection. Result demonstrated that the protein level of CDK2, CDK4, and CDK6 were fall off in miR‐524‐5p mimic group and raised in miR‐524‐5p inhibitor group (Figure 6c,d). siRNA‐ITGA3 group has similar pattern to miR‐524‐5p mimic group (Figure 6c,d). siRNA‐FOXE1 groups just partially downregulated the protein level of CDK2, CDK4, and CDK6 (Figure 6c,d).

## 4 DISCCUSION

Recent researchers identified more and more miRNA target on the PTC‐related genes, with the function on PTC development and progression through regulation of tumor cell proliferation, invasion, and migration. In general, miRNA differentially expressed in PTC tissue interacted with genes involved in cell cycle, apoptosis, and autophagy to promote or inhibit the related biological signaling pathway. However, few studies characterized the role of miR‐524‐5p on PTC. Thus, we analyzed miR‐524‐5p and its target genes *FOXE1* and *ITGA3* in PTC, and found miR‐524‐5p function as a PTC suppressor through reduction of PTC cell proliferation, invasion, and migration. Both *FOXE1* and *ITGA3* could be knockdown by miR‐524‐5p to enhance the PTC cell‐cycle arrest and apoptosis. The cell cycle and autophagy might be controlled by *ITGA3* and *FOXE1*, respectively.

First, two targets of miR‐524‐5p were identified in PTC. Dual‐luciferase reporter gene assays proved miR‐524‐5p could directly bind to 3′UTR of *FOXE1* and *ITGA3* (Figure 2). The *FOXE1* and *ITGA3* expression and protein levels were negatively associated with miR‐524‐5p expression in PTC cell lines, patients' tumor and papa‐cancer tissues (Figures 1 and 2b,c). There are several examples showed miRNA involved in cancer development by downregulation of multiple genes. miR‐451a could directly target on AKT1, MIF, and c‐MYC to reduce the signaling of AKT/mTOR pathway (Minna et al., 2016). In glioma, miR‐524‐5p could also directly knockdown expression of EZH2, Jagged‐1, and Hes‐1 (L. Chen et al., 2012; Zhi et al., 2017). Thus, our results confirmed that both *ITGA3* and *FOXE1* are targets of miR‐524‐5p in PTC development.

Second, miR‐524‐5p, *ITGA3*, and *FOXE1* established a suppression pathway on PTC development and progression. In this study, miR‐524‐5p, *ITGA3*, and *FOXE1* have been demonstrated to regulate PTC cycle arrest and apoptosis (Figure 5). Thus, the PTC tumor cell viability was controlled by miR‐524‐5p, *ITGA3*, and *FOXE1* (Figure 3). Also we detected regulation of PTC cell migration and invasion through miR‐524‐5p, *ITGA3*, and *FOXE1* (Figure 4). Combine with our clinical studies, TNM stage, degree of invasion, LNM, and degree of differentiation were associated with the expression level of miR‐524‐5p, *ITGA3*, and *FOXE1* (Table 1). ITGA3 has been identified as a factor to enhance cell proliferation and cell cycle in intrahepatic cholangiocarcinoma (Huang et al., 2018). ITGA3 could also play in bladder cancer to regulate cell apoptosis (Sakaguchi et al., 2017). In contrast, previous Genome‐Wide Association Study suggested that SNP in FOXE1 is strongly associate with the risk of PTC (He et al., 2015). Together, these results reveal a potential mechanisms underlying suppression of PTC progression through miR‐524‐5p, *ITGA3*, and *FOXE1* pathway.

Third, *ITGA3* or *FOXE1* might play in a different role during PTC development. In MTT assay, miR‐524‐5p mimic has more effect than RNAi–*ITGA3* or RNAi–*FOXE1* alone (Figure 3). The protein level of autophagy‐related protein LC3‐II, Bcl‐2, and BECLIN1 are changed under the regulation of miR‐524‐5p or *FOXE1* but not *ITGA3* (Figure 6). The effect of *FOXE1* on cell cycle might be attenuated that *ITGA3* (Figure 6). Recent studies reveal that the increase of FOXE1 is associated with autophagy markers such as LC3B, ATG5, ATG12, and BECLIN1 (Ji, Cui, Yu, & Cui, 2016). Taken together, *ITGA3*, and *FOXE1* might effect on cell cycle and autophagy, respectively.

In conclusion, FOXE1 and ITGA3 are direct targets of miR‐524‐5p in PTC. miR‐524‐5p could suppress PTC progression by regulating tumor cell proliferation, migration, and invasion. During the suppression, miR‐524‐5p downregulate FOXE1 and ITGA3 to enhance the PTC cell apoptosis. Also, PTC cell‐cycle arrest and autophagy might be controlled by miR‐524‐5p/ITGA3 and miR‐524‐5p/FOXE1, respectively.

## CONFLICT OF INTERESTS

The authors declare that there are no conflict of interests.

## AUTHOR CONTRIBUTIONS

Design and supervision of the study: J. Y. and S. J. Writing and the correction of the manuscript: H. L., X. C., J. Y., and S. J. Analysis of miRNA and identification of putative target genes: T. L. and X. C. qPCRs, luciferase assays were performed by H. L., X. C., T. L., and X. C. Transfected cell lines: T. L. and X. C. Statistical analysis: H. L. and X. C.
